# I *Feel* You: The Design and Evaluation of a Domotic Affect-Sensitive Spoken Conversational Agent

**DOI:** 10.3390/s130810519

**Published:** 2013-08-13

**Authors:** Syaheerah Lebai Lutfi, Fernando Fernández-Martínez, Jaime Lorenzo-Trueba, Roberto Barra-Chicote, Juan Manuel Montero

**Affiliations:** 1 School of Computer Sciences, University Science of Malaysia, Penang 11800, Malaysia; 2 Departamento de Teoría de la Señal y Comunicaciones, Universidad Carlos III de Madrid, Madrid 28911, Spain; E-Mail: ffm@tsc.uc3m.es; 3 Grupo de Tecnología del Habla, Universidad Politécnica de Madrid, Madrid 28040, Spain; E-Mails: jaime.lorenzo@die.upm.es (J.L.-T.); barra@die.upm.es (R.B.-C.); juancho@die.upm.es (J.M.M.)

**Keywords:** spoken conversational agents, affect prediction, domotic applications, affective agents, frustration, contentment, conversational features, user satisfaction, emotion model, emotion design and evaluation

## Abstract

We describe the work on infusion of emotion into a limited-task autonomous spoken conversational agent situated in the domestic environment, using a need-inspired task-independent emotion model (NEMO). In order to demonstrate the generation of affect through the use of the model, we describe the work of integrating it with a natural-language mixed-initiative HiFi-control spoken conversational agent (SCA). NEMO and the host system communicate externally, removing the need for the Dialog Manager to be modified, as is done in most existing dialog systems, in order to be adaptive. The first part of the paper concerns the integration between NEMO and the host agent. The second part summarizes the work on automatic affect prediction, namely, frustration and contentment, from dialog features, a non-conventional source, in the attempt of moving towards a more user-centric approach. The final part reports the evaluation results obtained from a user study, in which both versions of the agent (non-adaptive and emotionally-adaptive) were compared. The results provide substantial evidences with respect to the benefits of adding emotion in a spoken conversational agent, especially in mitigating users' frustrations and, ultimately, improving their satisfaction.

## 1. Introduction

Emotion is quintessential for intelligence, to the point that psychologists and educators have re-defined intelligence to include emotion and social skill. With the mass appeal of computer-mediated agents, computers are no longer viewed as machines whose main purpose is to complete tasks; rather, they are required to have the social abilities that humans naturally demonstrate in their daily interactions. Thus, the developments of spoken conversational agents (SCAs) typically move towards including socio-emotion content, which upgrades them to being socially intelligent.

This paper concerns the incorporation of a recently developed task-independent emotional model into a voice-only domotic agent, with the intention to upgrade the agent to be socially and emotionally intelligent. Using this model, the generation of emotion is driven by *needs*, inspired by human's motivational system, hence called NEMO (need-inspired emotion model). The intention is to incorporate NEMO into existing SCAs in order to enable them to be affect-sensitive. This is accomplished by predicting user affective states and responding to them with appropriate affective responses, through an emotional text-to-speech system. The focus of the paper is not on NEMO in itself (which has been described elsewhere [1]), but the value of the emotional intelligence equipped by this model when integrated in non-adaptive applications, in order to make them more adaptive to the users' emotion; specifically, whether an SCA that is affect-sensitive would reduce user frustration and increase user overall satisfaction of the SCA.

Though NEMO is a generic and task-independent architecture, actual events and situations are required in a specific domain in order to run this model. Therefore, to demonstrate affect sensing and generation through the use of this model, we describe the work of integrating this model with a natural-language mixed-initiative high-fidelity-control spoken dialog towards the goal of a socially intelligent HiFi agent. Specifically, this is described in the first part of the paper. The second part focuses on building a real-time automatic detection of affect, as robust automatic detection is vital to any affect-sensitive system. The final part concerns the user study conducted to compare both non-adaptive and adaptive agents.

## 2. Motivation and Related Work

Within the field of ambient intelligence (AmI), researchers are looking for ways to make agents adapt to users on the “fly”, either to the latter's emotions (as is the concern of this paper), preferences [2,3], age [4], environmental health [5] or, even, IQ [6]. The motivation is that an agent that adapts better to users would hopefully lead a more effective, empathetic and naturalistic interaction.

However, within spoken dialog systems, while there are considerable amounts of studies that address agent believability and, also, user affective states that accompany other environments, especially learning [7–11], games and entertainment [12–15] and call centers and information-services [16–18], very few aim at identifying emotions that influence interactions within a domestic environment. Studies in a closer domain, such as those of human-robot interaction (HRI) for intelligent homes, are typically concerned with the design space of service-robots towards improving their believability through life-like essences, such as appearance (e.g., anthropomorphism) and some other physical acts (e.g., head pose, gaze, motor skills), accounting for intimacy and engagement with the robot in order to increase people's acceptance of the former as companions [19,20]. However, the idea of demonstrating the social intelligence of a domestic agent in such a way that it would be regarded as a companion is quite far-fetched, considering the scope and the technical facet of the application (that may be a speech-only application) and the relevant affective states targeted for adaptation [8], which may be rather limited.

Thus, a plausible and feasible goal would be to have a dialog system that is expressive enough [21] that the human interlocutor responds to it by applying native speaker intuition. Though some users tend to treat machines as real humans [22], they may not mind some “hiccups” in the interaction, as long as there is no major breakdown in communication, as asserted by Edlund *et al*. [21].

## 3. Infusing Emotions into the HiFi Agent

We attempt to infuse emotions into an existing HiFi agent using NEMO. The baseline (non-adaptive version) HiFi system is a proprietary system developed by Grupo Technología del Habla (GTH), (see details in [23]). The HiFi agent controls and manages the HiFi audio system, and for end users, its functions equate to a remote control (select a CD, track or radio channel, record music, change channels, *etc*.), except that instead of clicking, the user interacts with the agent using voice.

As mentioned earlier, NEMO is a need-inspired system, whereby the agent elicits an emotion that is coherent with the situation *in view* of its different needs. One of the most influential needs for the agent's emotion modification is the Success need. Therefore, this section focuses on the Success need. The Success-need level is influenced by various events that are related to different tasks. Previously, this was done by updating a predefined percentage of an individual event (values differ according to events). For example, in the previous prototype of NEMO that was put to test with a domotic robot, Groucho, whose main tasks are to manage various domestic appliances (see [1,24] (a couple of demos showing applications of different domains used to test this model can be here: http://www.syaheerah.com/?page_id=789)), an event detected by sensory inputs, such as user touching or caressing the agent's face, might have a fixed value of 0.5, indicating a medium success level, or winning a game might have a fixed value of 0.7, a high success level. In integrating NEMO with the HiFi spoken conversational agent (henceforth “NEMOHIFI”), though, we moved a step ahead by adopting a more user-centric approach, using machine learning to *automatically predict* the said values by learning from past evaluation's data using a trained classifier, described later in Section 4. An interaction event is predicted as good or bad (and also *how* good/bad), and the corresponding values will then be taken to compute the Success need.

### 3.1. Architecture of NEMOHIFI

**Figure 1.**
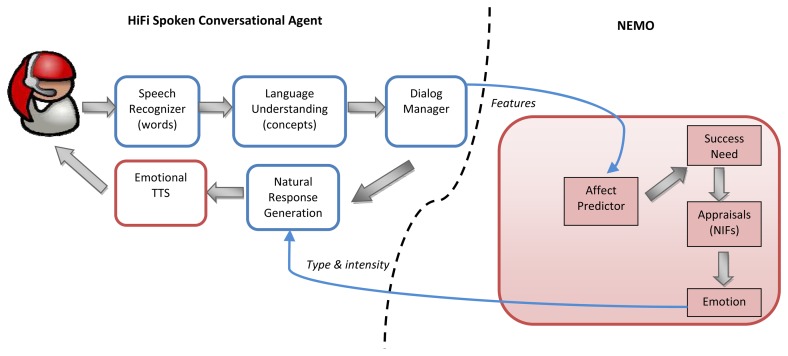
The architecture of the need-inspired emotion model with the HiFi spoken conversational agent (NEMOHIFI).

Most spoken dialog systems have an architecture that is similar to the HiFi SCA, as shown on the left side of Figure 1. The user utters a sentence, and the Speech Recognizer captures the sounds from the user's speech, matching the recognized words against a given set of vocabulary. Then, the matched words are passed to the Language Understanding module to extract the concepts (semantic information) of the sentence. A series of concepts are then passed to the Dialog Manager to activate dialog goals. The Dialog Manager decides both the actions to be taken and the feedback to the user for the current dialog turn and passes the semantic information to the Natural Response Generator module to generate a suitable textual response to the user. The text-to-speech (TTS) module then synthesizes the message and speaks to the user. The original non-adaptive HiFi SCA version used a neutral-voiced commercial TTS. A detailed architecture of the non-adaptive system is given in [23].

In converting the HiFi SCA into an affect-adaptive system, its existing components were *not* modified, except for the Natural Response Generation textual content (NRG). Instead, the HiFi SCA communicates *externally* with NEMO. The interaction between the system's modules and NEMO is shown in Figure 1. The information flow is similar, as described previously, but this time, the Dialog Manager additionally passes certain dialog features that are significant predictors of the user emotional state to the Affect Predictor. The Affect Predictor classifies the emotion state of the user following a Simple Logistics trained model. The classification result is then passed on to the need module to update the agent's Success need. Consider a user having a few bad dialog turns—perhaps the HiFi SCA failed to completely understand the user request and repeatedly asks the user to provide new information and extends the otherwise short dialog. In this case, the Dialog Manager sends certain relevant features (request turns, contextual information, *etc*.) to the Affect Predictor. Based on these features, the Affect Predictor predicts that the user is frustrated. This information is then updated to the need module, which modifies the agent's Success need. The agent now perceives the user as being frustrated, and therefore, its Success need is low. The dynamicity of the need level also depends on the situations of the previous turns; consecutive or continuous prediction that the user is frustrated causes the agent's Success need satisfaction to decrease rapidly, and so, when a good event (turn) appears right after (and the user is now predicted to be in a positive emotion), the agent will not immediately change its state to a joyful one, but rather surprised or neutral, depending on the situation. Conversely, if the agent is in a joyful state for sometime and continues with turns that are perceived as good (user predicted to be satisfied in consecutive turns), the drive to gratify its Success need will not be as significant as in the other case, and so, its joyful state reaches its maximum and starts decaying into a neutral state, though it continues to perceive the ongoing events as positive ones.

It should be noted, however, that the Dialog Manager receives dialog features only when the Speech Recognizer is successful in detecting the user's words. When speech recognition failure occurs, there will not be any executed actions, and this in itself is one of the features used to update the agent's Success need.

Next, the agent's Success need information updates the rest of the modules in NEMO and generates an emotion that is coherent with the agent's assessment of its current Success need. Finally, the chosen emotion matches against the natural response generation for a suitable response content and is synthesized into a speech response of a specific intensity of the chosen emotion by an Emotional TTS, known as the GTH-EMO TTS, built by [25]. GTH-EMO TTS is used in replacement of the original neutral one. It is capable of generating speech in various intensities of the Big Six emotional categories, proposed by [26]: neutral, joy, sadness, fear, anger, surprise and, also, a combination of these. This is done by adapting the vocal features and prosodical variation of the neutral emotion of a speaker into the same speaker's emotional voices. The affective voices have been studied through a perceptual test, which analyzed the emotion identification rates using neutral text (see [25]). Identification rates (in % accuracy) are 0.61 for neutral, 0.61 for joy, 0.65 for sadness, 0.78 for anger and 0.50 for surprise. Considering that these results are obtained using a neutral text (text that is not emotionally-inherent), we expect that identification rates for a real-time user study would be even higher, because the text involved would be emotionally-inherent (e.g., “I'm sorry, I am not doing a good job”, read in a sad way). Fear was not included in the perceptual test. However, not all of these emotions will be relevant in this study, as explained in Section 4.2.

## 4. Automatic Detection of Affect

Real-time automatic detection of emotion is vital to any affect-sensitive system. In this section, we describe the method used to automatically predict the agent's Success need value of NEMOHIFI, which will subsequently update the cognitive appraisals' module in order to generate a suitable affective response.

The descriptions of the procedures, methods and annotation protocols for efforts in automatic prediction of affect for this study are published elsewhere [27] (Readers are strongly recommended to read this paper in order to relate the efforts in automatic prediction of affect for this study with the user studies reported in the current paper).

As mentioned previously, in NEMOHIFI's context, the success rate of an interaction modulates the agent's need, particularly its Success need. An interaction is deemed successful when the *user* is content with the agent's performance. Thus, a fundamental challenge in converting a non-affective HiFi agent (or any systems in general) into an affective one is robust automatic detection of user affect. A highly satisfied user also satisfies the agent's success need; hence, user-agent satisfaction has a positive linear relationship. User affect can be reflected in the user *satisfaction* judgment [28–30], and the relationship of affect and satisfaction judgment has been empirically proven in [31,32] and, also, in our work, which will be further described.

To model user affect, we used *satisfaction rating* as the target and *conversational features* as predictors, obtained from a corpus collected in a past evaluation [33]. What makes our approach different from others is that we used target and predictor variables whose potentials are often ignored to model affect. While many studies focus on numerous channels for affect detection, very few have explored dialog as a potential source [7]. User affect could be mined from conversational elements, which are always cheaper and are usually obtained with little or no computational overhead. However, since the focus of this paper lies in modeling affect in the HiFi agent, we limit this section into summarizing the method and the outcome of the experiments carried out in automatic affect detection using the data from two studies, which will be described shortly.

### 4.1. User and Annotator Studies

To model affect by predicting user satisfaction, we used the HiFi-AV2 corpus, collected during a previous *user* study (see [34]). HiFi-AV2 consists of audiovisually recorded information of real, non-acted interactions (N = 190 interaction sessions) between users and the non-adaptive version of the HiFi agent. It is important to note that this user study was conducted with the intention of only measuring the agent's performance (*i.e*., ability to execute the actions requested by users), without foreseeing the integration of any social intelligence.

In this study, users interacted with the HiFi agent hands on, and at the end of each interaction, they rated the HiFi agent by providing a score between 1–5 Likert point (one, being very poor, to five, very good). Later, we used a reduced version of the same corpus to obtain satisfaction and affect-labeled data from several independent *annotators*. The corpus was reduced to 10 speakers that were chosen randomly (N = 100 sessions) to downsize manual labeling efforts. This study was similar to the first one, except that the annotators *also* perceived user emotion in each interaction—the annotators were given a set of full recordings (from the start until the end of an interaction), and they were free to label as many defined emotions (the nuances within the six basic emotions proposed by [26]) detected throughout the whole interaction. The annotators were asked to imagine themselves as users, and it is important to note that they were neither familiar with the users nor given any information about users' ratings, so as to not influence their own ratings. Thus, we could view both datasets as that of users' actual ratings and targeted ratings (by the annotator). Additionally, we also now have *affect-labeled* data by annotators.

### 4.2. Affective States Accompanying Interactions with Domestic Spoken Dialog Agents

Based on the observations of the interactions in the videos from past evaluations of the spoken dialog HiFi agent, we were able to identify a set of emotions that frequently occurred during user-HiFi agent interactions. Typical emotions involved were contentment, frustration, confusion and boredom. These emotions are within the same family of some of the basic emotions proposed by [26], namely, happiness, anger, surprise and sadness, respectively, but in finer and less intense nuances. One other emotion of interest was self-frustration, in which users displayed discontentment towards themselves for erroneously addressing the system. We also added neutral to represent situations where there was no particular emotion of the aforementioned type present.

This study would, however, focus on discriminating affect between two classes: contentment and frustration, two types of emotions that are known to be prevalent within spoken HCI. These two categories of affect represent positive and negative user emotional states and their varying intensities (e.g., at the end of an interaction, a particular user might have felt intensely content with the system when the user gave a score of five or “excellent” (on a five-point scale), and rather frustrated when he or she gave a score of three or “regular”), (depending on the model that was chosen—different models have different groupings of scores, elaborated later in Section 4.4. A score of three, for example, may either represent a low-intensity frustration (category 3, version 2) or slight contentment (category 3, version 1)).

The agent would respond to the user with a suitable emotion, reflected by its intensity and content, depending on the predicted user state. Specifically, the agent could speak in three different intensity levels of joy (low, medium, high) and two levels of sadness/regret (low, medium).

### 4.3. Affect Classification from Conversational Features

In order to obtain a model of user affect, we conducted two experiments; Experiment I was evaluated on the satisfaction-labeled data from both user and annotator subject groups. Experiment II involved the emotion-labeled data by annotators. In both types of experiments, we applied standard classification techniques in which several classifier schemes were utilized with the intention of comparing the performance of the various classification techniques, apart from determining which technique(s) yield the best performance. The Waikato Environment and Knowledge Analysis (WEKA) [35] was used for these purposes. One or more classification algorithms were chosen from different categories, including rule-based classifiers (ZeroR as the base rate (ZeroR predicts the class without considering other attributes, and only prior information from the training set.) and OneR), functions (SimpleLogistic, SMO), meta classification schemes (Multischeme, MultiBoost, AdaBoost) and trees (J48). A 10-fold cross validation technique was used for all the classification tasks.

### 4.4. Data Redistribution

All satisfaction-labeled datasets were first re-sampled in order to obtain a more uniform distribution; samples with similar outcomes were grouped together, and this was repeated five times to satisfy all combinations of classification problems, as shown in Table 1. This way, we were also able to determine which clusters obtained optimized classifications.

## 5. Summary of Results and Discussions

This section summarizes the results from the two experiments mentioned above.

### 5.1. Experiment I (Model 1)

Table 2 presents the statistically significant improvements of classification results over the base rate in percentage accuracy for Experiment I.

**Figure 2.**
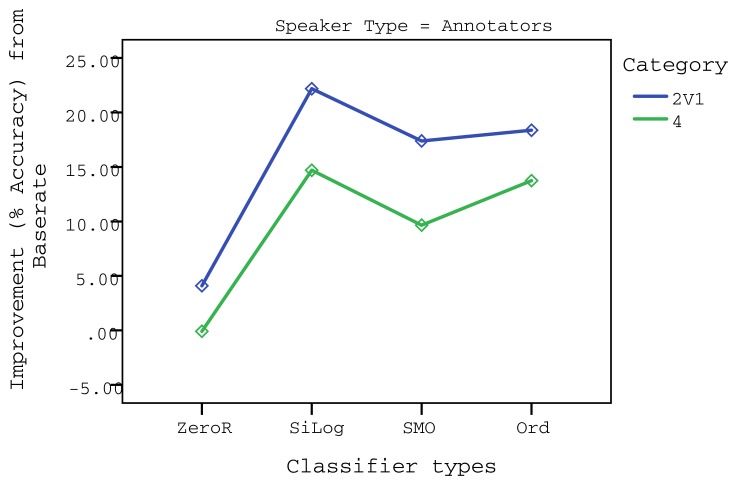
Summarized interaction chart for the annotators' dataset.

Results revealed that there was a significant effect of the subject type: *F*(1,40) = 83.07, *p* < 0.001, partial *η*^2^ = 0.68. Classifiers evaluated on user data mostly revealed worse results than the base rate, except SMO, whilst at least three classifiers that were evaluated on annotator data show significant improvement (at *p* < 0.001) over the base rate in each category, except categories 3V2 and 2V2. This indicates that most classifiers were able to predict satisfaction from dialog features based on the *annotator* data, suggesting that annotators were more impartial when judging the HiFi agent. Classification evaluated on annotator data yielded interesting result and, thus, is more suitable to be used for user affect modeling. Thus, we now focus on the results from annotator data. The chart in Figure 2 illustrates the interactions between the factors that obtained the best classification improvements for the *annotator* dataset, which are statistically significant.

### 5.2. Experiment II (Model 2)

Experiment II involved classification evaluated on the emotion-labeled data based on inter-annotator agreement. The computation that derive such agreement was adopted from [36]. Results revealed that the SMO scheme yielded the best statistically significant (at *p* < 0.01) improvement of classification over base rate, with improvement of 13.2%, followed by the Ordinal and Simple Logistics schemes, with 9.8% and 9.2% improvements respectively, both statistically significant at *p* < 0.05.

Both satisfaction and emotion-agreement data are significantly correlated (Pearson *r* = 0.29, at *p* < 0.01), but provide complementary information to model user affect. The data used for Model 1 involved users providing a satisfaction score at the end of each interaction; a global average score that represented the user's opinion of the agent—that could also be considered as the best representation of the score for each turn, should we have to rate the system on a turn basis. Thus, this information is suitable to be used to predict user affect on a *turn* basis. On the other hand, Model 2 involved annotators perceiving user emotion at different locations of a particular interaction, when the emotions displayed were most obvious. However, the information on the exact locations within the interaction was not noted. Hence, this kind of prediction is suitable to be performed at the end of the whole interaction, in order for the agent to be alerted that the user has been frustrated at some point during the interaction.

### 5.3. The Model Implemented in the Prototype

Although there was a possibility to use both models, as described above, for the NEMOHIFI real-time prototype, we only used the model based on the satisfaction-labeled data. We would need a simple affect prediction model in order to have a working real-time prototype. Considering that the difference in the means of the improvement rate between category 2V1 and 4 are not significant (as shown in Section 5.1), the first (two-class) classification model was chosen as the model for real-time affect detection for the affect-adaptable version of the HiFi agent (HiFi-NEMO) instead of the latter (four-class) model, to have a simpler classification output.

Upon implementing the Affect Predictor as part of NEMO in the NEMOHIFI prototype, we moved on to conduct a user study to test the value of NEMO through the prototype. The next section presents the experimental design, procedure and the subjects involved in the pilot study.

## 6. Experimental Set-Up

The main research question of this study is about the value of emotional intelligence in an SCA, using NEMO; specifically, whether an SCA that is affect-sensitive would reduce user frustration and increase user overall satisfaction of the SCA. To answer the research question, some changes were made to the HiFi agent. In order to determine the value of NEMO, we must identify which versions of the agent are preferred; the non-adaptive version (henceforth “BASEHIFI”) or the adaptive version, NEMOHIFI. A user study was conducted in a real-time environment to determine this.

### 6.1. Procedure

**Figure 3.**
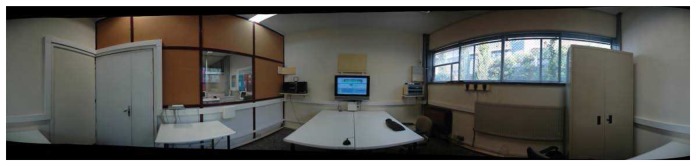
Demonstration room.

The experiment took place in the demonstration room of the Speech Technology Group, as shown in Figure 3. This room measures about 3.9 × 4.4 × 3 meters. The experimenter manages the evaluation from right outside of the room. The wall on the left-hand side of the room was covered with boards for two reasons: first, to limit the subject's visibility of the environment outside the room; and second, to help reduce users' anxieties, with the hope that by being more relaxed, they would behave more freely.

Subjects were asked to interact with both versions of the HiFi agent, approximately five minutes with each version. Six fixed scenarios were used to enable users to interact with each agent at least three times. In other words, the users were instructed to speak with the agent six times, in a random order, each for approximately five minutes. The time was controlled by the experimenter. After completing the consent form, subjects were briefed on the experiment. They were told that both versions of the agents use the same speech recognizer and synthesizer and have the same exact functionalities. They were also told that the scenarios were only guidelines to scope out the possible tasks that the agent could address and that they were free to go ahead with tasks beyond the recommended scenarios, but within the functionalities of the HiFi agent. It is important for readers to note that this experiment is different from the previous one mentioned earlier [34], as we were interested in the social part of the agent that impacts user satisfaction and not in its performance (e.g., the ability to understand users accurately). Thus, instead of strictly asking the users to abide by the given guidelines in terms of scenarios (mission-based), users were encouraged to address the system as they wish within the given five minutes, as long as their requests are within the system's functionality. This way, users would not be concentrating so much on whether or not the agent has finally addressed the user's request, but, instead, would focus more on the behavior of the agent in reaching towards a particular goal.

At the end of the evaluation, the experimenter interviewed the user on his/her opinion of both agents. Finally, the user was asked to annotate two of his/her interactions on a turn basis, on an Excel sheet, while watching his or her own recorded video. Among others, the self-turn-based emotion annotation would be especially useful for future research.

### 6.2. Subjective Metrics

**Figure 4.**
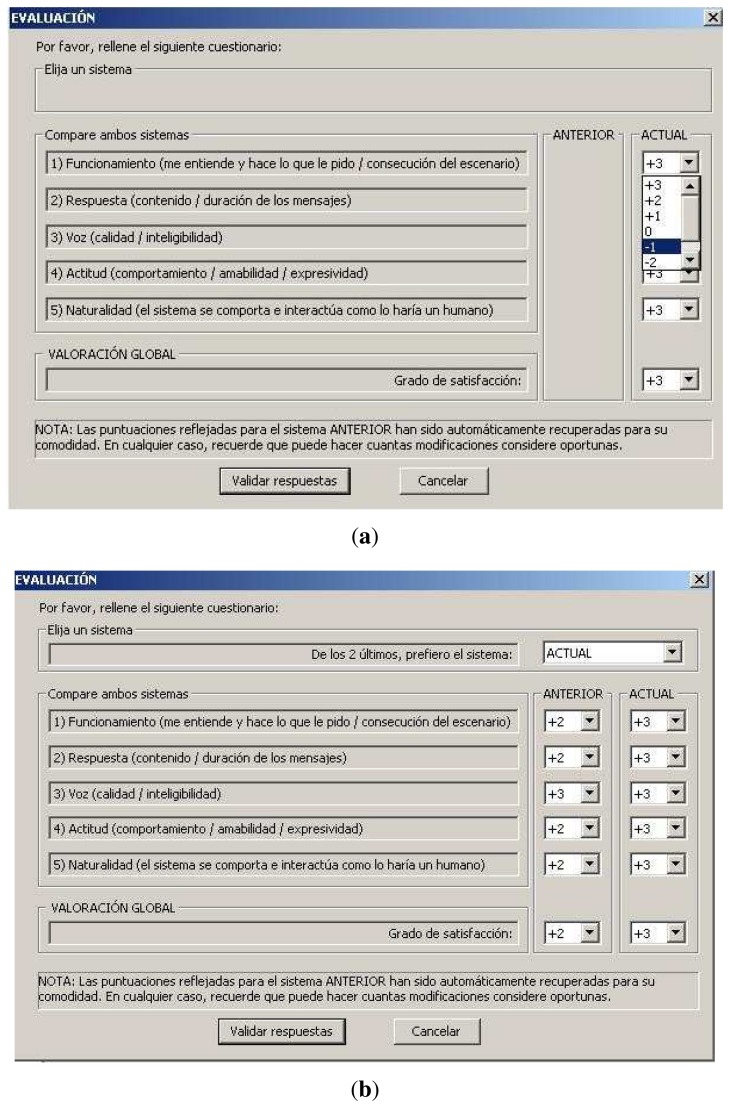
The virtual questionnaires. (**a**) Ratings for the current agent; (**b**) ratings for the next agent, along with the reference of the previous one, in which users were allowed to edit.

After each interaction session, users were asked to evaluate the agent by filling out a questionnaire that was displayed virtually on the television screen. We adopted a 7-point Likert scale, from +3 (excellent) to −3 (very poor), instead of a 5-point one, as was used in the previous user/annotator study (see [33]). The main reasons for this were that a scale with more response categories would transmit a greater amount of information, and a 0-centered scale would enable users to better distinguish positive categories from the negative ones (e.g., +1 to +3 as positive and *vice versa*), thereby providing more reliable feedback. The virtual questionnaire is presented in Figure 4 below. The following is the translated questions into English:

1. FUNCTIONALITY/PERFORMANCE—The system understands me and performs my requests accordingly.
2. RESPONSE—Content/duration of the messages.
3. VOICE—The TTS quality and intelligibility.
4. ATTITUDE—The system's behavior/friendliness/kindness/expressivity.
5. NATURALNESS—The system behaves and interacts as a human would.
6. OVERALL SATISFACTION—The overall satisfaction, taking into account all the metrics above.

Additionally, after interacting with the second agent onwards, subjects were asked to select their *preference* (PREFERENCE) between the current and previous system in the virtual questionnaire (see Figure 4b). As can be observed, subjects were given the chance to make *explicit comparisons* between pairs of systems—that is, to refer and edit their ratings of a particular agent based on the ratings of its pair.

### 6.3. Subject Pool

The subjects involved in the user study were undergraduate and postgraduate students, between 18 to 35 years old, mostly from Universidad Politécnica de Madrid. Twenty-four subjects participated (11 males and 13 females)m and none of the subjects had interacted with the HiFi agent before the experiment. The subjects were offered incentives in the form of cash for participation.

## 7. Results

**Figure 5.**
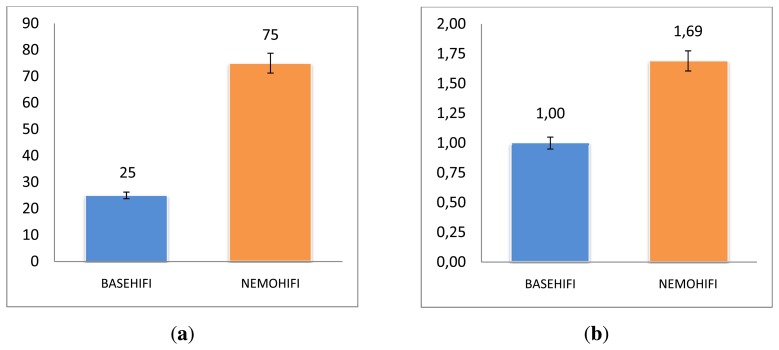
Comparisons of preference and satisfaction ratings between BASEHIFI and NEMOHIFI. (**a**) Preference percentage for both agents; (**b**) mean satisfaction ratings for both agents.

- **User preference**: Out of 72 pairs of interactions (N = 72 [three pairs × 24 subjects]), 25.0% preferred BASEHIFI and 75.0% opted for NEMOHIFI, as shown in Figure 5a. There was a highly significant association between user preference and agent type, χ^2^(1) = 36.0, *p* = 0.000. This seems to represent the fact that based on the odds ratio (the odds ratio is calculated this way: number of positive outcomes/number of negative outcomes of Group I (50/22—for NEMOHIFI) divided by the number of positive outcomes/number of negative outcomes of Group 2 (22/50—for BASEHIFI); [37]), the subjects would be 9.1 times more likely to prefer the NEMOHIFI agent than the BASE one.
- **Comparisons of subjective metrics between agents**: Table 3 shows the comparisons of means between both agents and whether they were statistically significant (denoted by *) according to the paired *t*-test. The *t*-test revealed that:
  – there were no significant differences in the perceptions of PERFORMANCE and RESPONSE.
  – on average, VOICE was rated significantly higher for the NEMOHIFI agent (*M* = 2.14, *SD* = 1.24) than the BASEHIFI one (*M* = 1.67, *SD* = 1.14, *t*(7) = −3.31, *p* = 0.001), with a fairly substantial effect size (*r* = 0.36).
  – on average, ATTITUDE was rated significantly higher for the NEMOHIFI agent (*M* = 2.11, *SD* = 1.27) than the BASEHIFI one (*M* = 0.90, *SD* = 1.33, *t*(71) = −7.76, p = 0.000), with a highly substantial effect size (*r* = 0.68).
  – on average, NATURALNESS was rated significantly higher for the NEMOHIFI agent (*M* = 1.54, *SD* = 1.42) than the BASEHIFI one (*M* = 0.42, *SD* = 1.31, *t*(71) = −7.41, *p* = 0.000), with a highly substantial effect size (*r* = 0.67).
  – most importantly, on average, the OVERALL SATISFACTION was rated significantly higher for the NEMOHIFI agent (*M* = 1.69, *SD* = 1.27) than the BASEHIFI one (*M* = 1.00, *SD* = 1.16, *t*(71) = -4.89, *p* = 0.000), with a highly substantial effect size (*r* = 0.50)—see Figure 5b.
- **Comparisons of objective metrics between agents**: Table 4 shows the differences in certain objective metrics between both agents that were significant, namely, average sentence recognition confidence (Ave_sentence_recog_conf), executed actions (Exec_action) and turns taken (TT). All of values for these metrics were significantly higher for BASEHIFI than for NEMOHIFI, as reported below:
  – on average, there was significantly higher Ave_sentence_recog_conf for BASEHIFI (*M* = 0.75, *SD* = 0.76) than NEMOHIFI (*M* = 0.73, *SD* = 0.09, *t*(71) = 2.13, *p* = 0.04, *r* = 0.25).
  – on average, there was significantly higher Exec_action for BASEHIFI (*M* = 30.47, *SD* = 8.3) than NEMOHIFI (*M* = 27.7, *SD* = 7.8, *t*(71) = 2.77, *p* = 0.01, *r* = 0.31).
  on average, there were significantly higher TT for BASEHIFI (*M* = 21.79, *SD* = 3.96) than NEMOHIFI (*M* = 19.97, *SD* = 4.15, *t*(71) = 3.52, *p*, = 0.001, *r* = 0.39).
- **Gender effect**: The MANOVAsimple-effect analysis shows that there was a significant interaction effect between user gender and the perception of RESPONSE of the NEMOHIFI agent, in which the female subjects gave a significantly higher score (*M* = 1.60, *SD* = 0.1) than male subjects (*M* = 0.94, *SD* = 1.60) at *p* = 0.03.

### 7.1. Discussion on the Results

The results above revealed that users would be more likely to select the NEMOHIFI agent three times more over the BASEHIFI, (a staggering nine times when analyzing the odds ratio). Moreover, over 80% of the users (20 out of 24) selected the NEMOHIFI agent as their preference after interacting with both agents for the first time (the first pair). The first was also perceived to be substantially significantly better in its voice, attitude and naturalness. More importantly, users were overall highly more *satisfied* with the NEMOHIFI version than the BASE one.

The response of the BASEHIFI agent was found to be slightly better than the NEMOHIFI, and this was not that surprising, as it could be due to the fact that the first had limited variation of prosody, clearer pronunciation and shorter duration. A similar finding was found in [38]. The NEMOHIFI version's response (NRG content) was considerably lengthier, and the addition of prosody has made it even more lengthy than in previous experiments. Additionally, due to this fact, the turns taken were significantly less, as users were given less chance to speak. However, while, in general, the NEMOHIFI agent's response was slightly worse than BASEHIFI, it was significantly appreciated by female subjects when compared to males. This was consistent with the majority of the female subject's comments—that the NEMOHIFI agent was “sensitive” and much more “kinder” compared to the BASEHIFI. This shows that female users in this study tended to be more easily affected by the content of the speech.

There were no noticeable differences perceived between both agents' performances, although in reality, it was otherwise. Table 4 shows that the NEMOHIFI agent was significantly performing worse than the BASEHIFI, suggested by significantly lower dialog average recognition confidence, achieved goals and, most importantly, efficiency (based on the percentage of executed actions). However, NEMOHIFI was still significantly preferred and scored higher satisfaction ratings. These findings could be explained in part by the way users communicated to the agents. As per the experimenter's observation, users *adjusted* their ways of communicating to the agents depending on the behavior of the agent. On one hand, users tended to address the NEMOHIFI agent in a more spontaneous, human-like manner (*i.e*., smalltalk), and as such, the NEMOHIFI agent was not able to cope with recognizing all the words. On the other hand, users used more one-word machine-talk with the BASE agent, after realizing that it was not as natural as the first. However, more importantly, while in this study, examining the objective metrics was not the main concern, this serendipitous discovery paves the way for further research for exploitation of emotions as *compensation* to lower performance of an SCA in a similar domain.

To conclude, these substantial findings not only show that NEMO has successfully demonstrated its functions, but also unveil that the addition of emotion with adequate intensities improves not only user satisfaction, but also most of the other social qualities, such as voice, attitude and naturalness of the SCA.

## 8. Conclusions

In this paper, we proposed an approach of incorporating emotions into spoken conversational systems using NEMO. We demonstrated this by describing the integration on a proprietary baseline system, a non-adaptive HiFi agent. NEMO was then evaluated through the HiFi agent, specifically to assess the effects of the affective detection and reactions of the system. To conclude, we list several contributions of this paper, as the following:

- Our main contribution is to show empirically that NEMO has successfully demonstrated its function as a task-independent emotion model, at least in the domain presented in this paper.
- Our second contribution is to show empirically that infusing emotional intelligence into an SCA can mitigate user frustration, positively affect user perception of the SCA and ultimately lead to significantly higher satisfaction. Interestingly, users still preferred the adaptive agent to the non-adaptive agent, although the first had performed worse than the latter.
- Our third contribution is to show that infusing affect in the HiFi agent was established without modifying any performance-related modules in the system (e.g., dialog manager, speech recognition, natural language understanding, *etc*.). Specifically, the Dialog Manager of the baseline system was neither modified nor hardwired with affect-related rules, as is done in most existing dialog systems, in order to be emotionally rich. Instead, the Dialog Manager communicates with the emotion system and manages the dialog using the emotionally-relevant features provided by the emotion classifier. Additionally, the emotion classifier is based on a learning-by-example method (of past data), not an imperative, hand-crafted one. These minimize costs in two ways; first, not only the requirement for domain-specific expert knowledge can be reduced, but the adaptation is also more user-centric. Second, this model could also be re-used in new, but similar domains, with minimum labor.
- Our final contribution is to show empirically that conversational features, a non-conventional source, could be used as a single source to model user affect reliably by predicting satisfaction ratings, however, within a limited-task domestic domain. The conversational features were the predictors, and the satisfaction judgments were the target. For this task, we used an annotation method that is less sophisticated (such as the use of untrained judges to rate both satisfaction judgments and emotions) and a smaller array of features for classification tasks. Nevertheless, emotion classification improvements achieved statistically significant results over the base rate.

## 9. Future Directions

Considerably more work will need to be done to improve the NEMO model. Our future efforts would focus on the following;

- as discussed in Section 6.1, users were asked to self-annotate their own recordings. It would also be interesting to run individual classification experiments using both satisfaction-labeled data from the *self-annotation* corpus (turn-based) and satisfaction-labeled data from the agent-user *interaction* corpus (dialog-based) and compare both results to check for any possible bias in ratings, as we have done previously (as shown in the user/annotator comparative results in Section 5.1).
- the finding that provides an insight with regards to using emotions as a compensation to low-performance in an SCA, as mentioned in the user study discussions (Section 7.1), should be investigated further. This would require a careful design for an experiment comparing a low-performing SCA with emotions and a better-performing one without emotions, in order to confirm the hypothesis that a worse-performing agent could be compensated by the exploitation of emotions.

Finally, we hope that this paper presents a strong case for the nexus of spoken conversational agents and emotions, but more importantly, that it provides a platform for potentially fruitful future research in this burgeoning area.

## Figures and Tables

**Table 1.** Datasets re-clustered according to similarity of score into all possible combinations of classes.

**Category**	**Label**				

	**Very Poor**	**Poor**	**Satisfactory**	**Good**	**Excellent**
Five ((5) original class)	1	2	3	4	5
% distribution (U, A)	2, 1	5, 16	22, 36	33, 29	38, 18
Four (4)	-	1, 2	3	4	5
% distribution (U, A)		7, 17	22, 36	33, 29	38, 18
Three (version 1)(3V1)	-	1, 2	3	4, 5	-
% distribution (U, A)		7, 17	22, 36	71, 49	
Three (version 2)(3V2)	-	1, 2, 3	-	4	5
% distribution (U, A)		29, 53		33, 29	38, 18
Two (version 1) (2V1)	-	1, 2, 3	-	4, 5	-
% distribution (U, A)		29, 53		71, 47	
Two (version 2)(2V2)	-	1, 2	-	3, 4, 5	-
% distribution (U, A)		7, 17		93, 83	

**Table 2.** Comparisons of significant improvements in classification accuracies in detecting the satisfaction score from conversational features (for both the *user* and *annotator* datasets).

**Category**	**Classifiers**							

	**Base Rate**		**SiLog**		**SMO**		**Ord**	
	**U**	**A**	**U**	**A**	**U**	**A**	**U**	**A**
Five	38.0	36.0	-	49.3	-	44.6	-	51.3
Four	38.0	36.0	-	53.1	-	43.4	-	52.0
Three (version 1)	71.0	47.0	-	64.0	-	61.1	-	62.5
Three (version 2)	38.0	53.0	-	-	50.7	-	-	-
Two (version 1)	71.0	53.0	-	75.0	-	74.4		69.4
Two (version 2)	93.0	83.0	-	-	-	-	-	-

SiLog = Functions.SimpleLogistics, SMO = Functions.SMO, Ord = Meta.Ordinal. U = user data, A = annotator data. Results were truncated to display only the best statistically significant classification improvements (at *p* < 0.05).

**Table 3.** *t*-test results comparing the mean subjective ratings between BASEHIFI and NEMOHIFI.

**Metric**	**Mean**		**BASEHIFI–NEMOHIFI**	***t***	**Sig.**

	**BASEHIFI**	**NEMOHIFI**			
PERFORMANCE	1.13	1.41	−0.28	−1.43	0.16
RESPONSE	1.46	1.29	0.17	1.05	0.30
VOICE	1.67	2.14	−0.47	−3.31	0.001 *
ATTITUDE	0.90	2.11	−1.21	−7.76	0.000 *
NATURALNESS	0.42	1.54	−1.32	−7.41	0.000 *
GSS	1.00	1.69	−1.21	−4.89	0.000 *

* denotes highly significant result at *p* < 0.001.

**Table 4.** The objective metrics with significant differences between BASEHIFI and NEMOHIFI.

**Metric**	**Mean**		**BASEHIFI–NEMOHIFI**	***t***	**Sig.**

	**BASEHIFI**	**NEMOHIFI**			
Ave_sentence_recog_conf	0.75	0.73	0.02	2.13	0.04 *
Exec_actions	30.47	27.68	2.79	2.77	0.01 *
TT	21.79	19.97	1.82	3.52	0.001 *

* denotes significant result at *p* < 0.05.
